# New Biodegradable Copolymers Based on Betulin and Hydroxycarboxylic Acid Derivatives

**DOI:** 10.3390/ma17050981

**Published:** 2024-02-20

**Authors:** Alexey Zinovyev, Alina Gorbunova, Anna Chernova, Sónia A. C. Carabineiro, Ekaterina Poletykina, Anastasia Bugaeva, Viktor Novikov, Ekaterina Kolobova, Alexey Pestryakov

**Affiliations:** 1 Research School of Chemistry and Applied Biomedical Sciences, National Research Tomsk Polytechnic University, Lenin Av. 30, 634050 Tomsk, Russia; alexzin1243@gmail.com (A.Z.); aag84@tpu.ru (A.G.); e.y.poletykina@gmail.com (E.P.); vikt46@yandex.ru (V.N.); 2 School of Earth Sciences and Engineering, National Research Tomsk Polytechnic University, Lenin Av. 30, 634050 Tomsk, Russia; apa@tpu.ru (A.C.); aab177@tpu.ru (A.B.); 3 LAQV-REQUIMTE, Department of Chemistry, NOVA School of Science and Technology, Universidade NOVA de Lisboa, 2829-516 Caparica, Portugal; sonia.carabineiro@fct.unl.pt

**Keywords:** renewable raw materials, betulin, biodegradable polymers, polycondensation, ROP

## Abstract

In this study, we propose an approach to the synthesis of new biodegradable polymer materials based on renewable raw feedstock (betulin) and derivatives of hydroxycarboxylic acids using a catalyst/catalytic system (γ-Al_2_O_3_, γ-Al_2_O_3_/TBHP) that is safe for health and the environment. The resulting polymers are linear thermoplastic polymers that undergo collapse upon melting in the presence of atmospheric oxygen. Moreover, these polymers demonstrate non-toxicity towards a range of Gram-positive and Gram-negative bacteria. The polycondensation of betulin with butyl lactate is particularly noteworthy.

## 1. Introduction

In recent years, the growth rate of polymer material production has steadily increased. Simultaneously, there is the pressing issue of recycling polymer waste after the expiration of the service life of materials and products derived from them. This challenge can be addressed by developing a new generation of polymer materials that combine the advantages of industrially produced synthetic polymers with the environmentally friendly characteristics of naturally occurring polymers capable of biodegradation.

One actively developing area in the field of biodegradable polymers involves the use of biomass or its processed products as feedstock. Betulin (lup-20(29)-ene-3,28-diol, C_30_H_50_O_2_, CAS: 473-98-3, Figure 1) is one of those products. It is a naturally occurring pentacyclic triterpene non-aromatic dihydric alcohol found in birch bark (up to 35%). Betulin exhibits broad-spectrum biological activity (including immunostimulating, antioxidant, hepatoprotective, anti-inflammatory and antiviral properties) [1,2]. Despite being a non-aromatic compound, the betulin molecule has an unusually high melting point of 256 °C due to its rigid triterpene framework consisting of 30 aliphatic carbon atoms. The betulin molecule has three functional groups: primary C-3 and secondary C-28 hydroxyl groups and an isopropenyl C-19 group. These three reaction centers allow the obtaining of a wide range of compounds with more pronounced biologically active properties through the chemical transformation of betulin. In addition, being a diol, betulin can participate in polycondensation and copolymerization reactions.

Beyond its biologically active properties and low toxicity, betulin has a bulky cycloaliphatic structure that can confer rigidity and thermal stability to polymers. In addition, its chemical transformation, including polymerization, addresses a key challenge by preventing the creation and widespread use of therapeutic drugs based on betulin—its insolubility in most biological fluids.

Thus, taking into account that a significant portion of birch bark is a by-product of the wood processing industry [3], betulin is undoubtedly an underutilized natural resource. It has the potential to yield not only very valuable biologically active substances but also a wide range of new biodegradable polymer materials characterized by biocompatibility, low toxicity and excellent operational characteristics.

At the same time, there are a limited number of studies dealing with the use of betulin as a monomer for polymer synthesis [4,5,6,7,8,9,10,11,12,13,14,15,16,17,18,19,20]. Table 1 shows some examples. Nevertheless, the authors of these studies note that polymer materials based on betulin can be used for various purposes, such as polymer binders for thermoplastic composite materials [17], polymer materials for gas sorption [9], modification of cellulose fibers [10,11], production of thermoplastics and thermosets [13,14,15,16,18,19,20], as well as the creation of biocompatible multifunctional biomaterials and carriers for controlled drug delivery and release [4,5,6,7,8,12].

In many of the cited works, despite achieving copolymer yields of 70–92%, an additional stage is required to obtain a betulin-based monomer, or dicarboxylic acid dichlorides are employed as comonomers, with organometallic compounds and pyridine acting as copolymerization catalysts. However, these substances are toxic and need specific precautions during use. Moreover, their separation and neutralization are time-consuming and resource-intensive processes.

In this regard, the purpose of this work is to demonstrate the possibility of producing betulin copolymers with biocompatible acids and their derivatives using a catalyst that is safer for health and the environment. In addition, this study aims to characterize the resulting polymers and assess their toxicity.

## 2. Experimental Section

### 2.1. Materials

Betulin > 95% (Betulika Co., Ltd., Yekaterinburg, Russia), butyl lactate 97% (Merck, Darmstadt, Germany), lactic acid (80% aqueous solution, Purac Corbion, Blair, NE, USA), glycolic acid 67% aqueous solution (Acros Organic, Geel, Belgium), L-lactide 98% (Acros Organic, Geel, Belgium), glycolide 99% (Acros Organic, Geel, Belgium) and adipic acid 98% (Merck, Darmstadt, Germany) were used as comonomers. Betulin was dried under vacuum at 50 °C. L-lactide and glycolide were recrystallized in toluene. Chloroform (HPLS grade, Merck, Darmstadt, Germany) and heptane (HPLS grade, Merck, Darmstadt, Germany) were used for polymer reprecipitation. γ-Al_2_O_3_ (Versal gamma alumina (VGL-25), UOP, New York, NY, USA), Sn(Oct)_2_ (Merck, Darmstadt, Germany) and Zn(OAc)_2_ (Merck, Darmstadt, Germany) were used as catalysts. Tert-butyl hydroperoxide (70% TBHP, Merck, Darmstadt, Germany) was used as a cocatalyst. Dimethyl sulfoxide (DMSO) (99.8% grade, PanReac Applichem, Barcelona, Spain) was used to prepare the samples for the disk diffusion test.

The structural and textural properties of γ-Al_2_O_3_ were investigated in a previous work [21] and are presented in Table 2.

All used strains of microorganisms were provided by the All-Russian collection industrial microorganisms National Research Center “Kurchatov Institute” (Moscow, Russia).

### 2.2. Methods

Betulin-based polymers were synthesized using adipic acid (AA), lactic acid (LA), glycolic acid (GA) and hydroxy acid derivatives, including lactide (Lac), butyl lactate (BL) and glycolide (Glc). The molecular ratio of betulin to acids for synthesis was 1:1. The synthesis was carried out in a single-neck round-bottom flask at a temperature of 180 °C with constant stirring under an argon inert atmosphere and a residual pressure of 7 mbar for 3 h, 15 h and 24 h, respectively. γ-Al_2_O_3_ was used as a catalyst at 0.1 wt.%, and tert-butyl hydroperoxide (TBHP) was used as a cocatalyst at a concentration of 0.1 mol.%. After synthesis, the polymers were dissolved in chloroform at a concentration of 5 wt.% for 2 h at room temperature and reprecipitated in fivefold hexane. The powder was dried at low pressure (7 mbar) and 35 °C for 24 h.

The catalytic activity of the proposed system was compared with an equivalent concentration of Sn(Oct)_2_, Zn(OAc)_2_ and without a catalyst.

^1^H NMR analyses were conducted using a Bruker AVANCE III HD 400 MHz spectrometer (Bruker BioSpin GmbH, Rheinstetten, Germany) at 25 °C in deuterated chloroform (CDCl_3_) at a concentration of 40 mg mL^−1^. The identification of the betulin conversion products was carried out by NMR spectroscopy in a previous paper [22].

Polymer molecular weight was determined in chloroform by gel-permeation chromatography (GPC) using an Agilent Technology 1260 chromatograph equipped with an Agilent PLgel 5μm Mixed-C chromatography column (300 × 7.5 mm) and a refractometric detector. A 20 μL loop was used for sample injection. It was calibrated with Agilent EasiVial PS-M polystyrene standards. Amylene-stabilized chloroform was used as the mobile phase. The polymer concentration in the analyzed sample was 1 mg·mL^−1^.

Thermogravimetry measurements were performed on a TA Instruments SDT Q600 (TA Instruments, Crawley, UK) by heating the sample from room temperature to 300 °C at 10 °C min^−1^ in air using a corundum crucible. Differential scanning calorimetry measurements were performed on a TA Instruments DSC Q2000 (TA Instruments, Crawley, UK) by heating the sample from room temperature to 275 °C at 10 °C min^−1^ in air using an aluminum crucible.

Polymer films were prepared by solvent casting concentrated solution (TCM, 10 gL^−1^) on a glass Petri dish and allowing it to stand for 48 h at ambient pressure and room temperature. The film was then removed from the surface and cut with a razor blade. The typical sample size was 10 × 10 × 0.090 mm.

The toxicology of polymers was studied using the disk diffusion method on an agar nutrient medium in Gram-positive and Gram-negative cultures. The Gram-positive strains *Staphylococcus aureus* B-664 (*S. aureus*), *Lactobacillus plantarum* 8P-A3 (*L. plantarum*) and *Staphylococcus epidermidis* PCI 1200 (*S. epidermidis*), and the Gram-negative strain *Escherichia coli* B-11333 (*E. coli*) were chosen as tested strains due to their different cell wall structures and presence in human microflora.

Cultivation of *E. coli* involved a dense nutrient medium, Luria–Bertani (LB), incubated at 37 °C for 24 h using the Precise Shaking Incubator (WiseCube WIS-20, Daihan, Seoul, Republic of Korea). Strains *S. aureus* and *S. epidermidis* were cultivated in a liquid nutrient medium beef-extract broth by an orbital shaker, the Precise Shaking Incubator (WiseCube WIS-30, Daihan, Seoul, Republic of Korea) at 37 °C, 100 RPM and 24 h. *L. plantarum* was cultivated in a Moser–Rogosa liquid nutrient medium using the Precise Shaking Incubator at 37 °C, 100 RPM and 20 h.

Polymer films were prepared in a solvent solution (DMSO) on a glass Petri dish and allowed to stand for 24 h at room temperature. The tested sample size was 10 mm in diameter.

A suspension of bacterial species (*E. coli*, *S. epidermidis, S. aureus*) with a concentration of 3 units, according to the McFarland turbidity standard, was prepared to be used in further tests. One day before the antibacterial activity test, a 1% agar solid nutrient medium was prepared, poured into Petri dishes (D = 90 mm) and incubated for 24 h in a thermostat at 28–30 °C to ensure sterility. For the disk diffusion method, 300 μL of each bacterial culture suspension was applied to the agar surface and evenly spread over the surface with a Drigalski spatula. Samples under investigation were placed on the dried nutrient medium using tweezers (the treated side was placed on the surface of the nutrient medium). Finally, the Petri dishes with samples were incubated in the Precise Shaking Incubator (WiseCube WIS-20, Daihan, Republic of Korea) at 37 °C for 48 h. The toxicology of the samples was determined in transmitted light by estimating the width of the inhibition zone of bacterial growth from the edge of the sample. The results were expressed in mm and presented as the mean ± SD.

A 500 μL suspension of Bacillus *L. plantarum* with a concentration of 3 units, according to the McFarland turbidity standard using a dosator, was placed in 4500 μL of melted (45–50 °C) sterilized dense Moser–Rogosa nutrient medium (MRS) with crushed polymer films and cultivated in an incubator (Precise Shaking Incubator, WiseCube WIS-20, Daihan, Seoul, Republic of Korea) at 37 °C for 72 h. Moreover, we also investigated the toxicity of polymer samples from the culture of *Lactobacillus plantarum* in a liquid nutrient medium using spectrophotometry. The experiments were carried out in 8 test tubes of 10 cm^3^. For the cultivation of bacteria, liquid nutrient media MRS containing polymer samples Glc and BL at a concentration of 1% (wt.) were used; a control medium without polymer samples was also used. Polymer samples were introduced during medium preparation and autoclaved in a WiseCube autoclave at 121 °C for 20 min. A 3% (vol.) 20-h culture of *L. plantarum* was used as the inoculum (seed material). After inoculation, the cultures were incubated for 34 h at 37 °C with a stirring speed of 80 rpm in the CO_2_ incubator (Thermo Scientific, Waltham, MA, USA). Bacterial biomass increase was determined through a spectrophotometric method. The change in the optical density of the bacterial suspension was measured at intervals of 0, 4, 8, 12, 24, 28, 32 and 34 h of bacterial cultivation by using a UV-VIS spectrophotometer Cary 60 (Agilent Technologies, Santa Clara, CA, USA) at a wavelength of 540 nm and a cuvette thickness of 1 cm. Based on the optical density data, a graphical dependence was constructed in the coordinates A/A_0_—time, where A is the optical density at the current time and A_0_ is the optical density at time 0.

## 3. Results and Discussion

For the first step, it was necessary to evaluate the principal possibility of the copolymerization of betulin with organic acids using γ-Al_2_O_3_ as a catalyst. For this purpose, a one-stage, 3 h synthesis was carried out with different biocompatible acids, namely adipic acid (AA), lactic acid (LA) and glycolic acid (GA). The obtained samples were further examined by GPC. The values of M_n_^GPC^ and M_w_^GPC^ calculated from the data of GPC are presented in Table 3.

**Figure 2:**
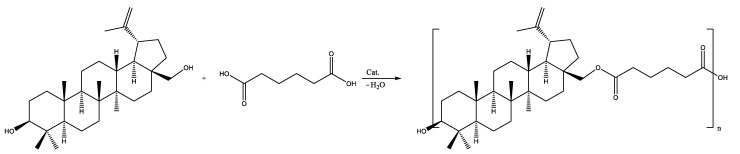
Polycondensation of betulin with AA.

In the case of the copolymerization of betulin and AA (Figure 2), a copolymer with a number-average molecular weight of 1600 g·mol^−1^ was obtained (Table 3). This value is in good agreement with the values of M_W_ presented in the literature [14] for the copolymerization of betulin and AA (1400 g·mol^−1^). The structure of the copolymer was confirmed by ^1^H NMR (see below).

**Figure 3:**
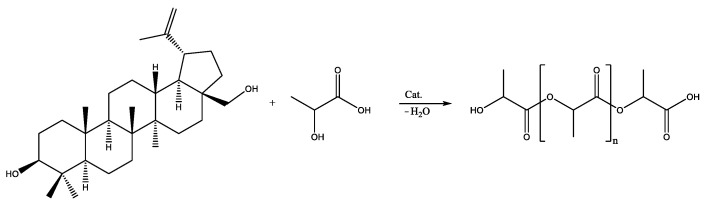
Polycondensation of betulin with LA.

**Figure 4:**
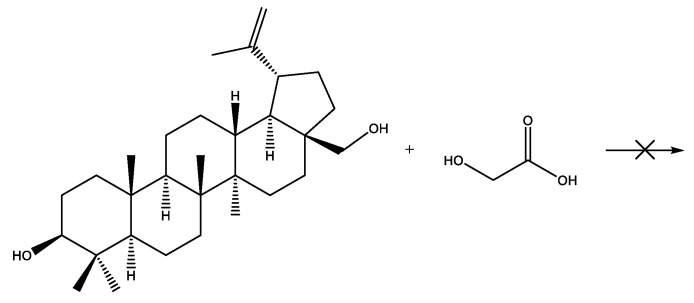
Polycondensation of betulin with GA.

**Figure 5:**
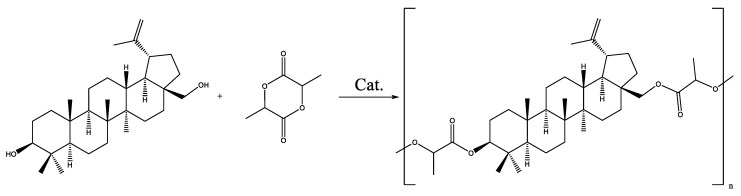
Formation of betulin copolymer with Lac.

**Figure 6:**
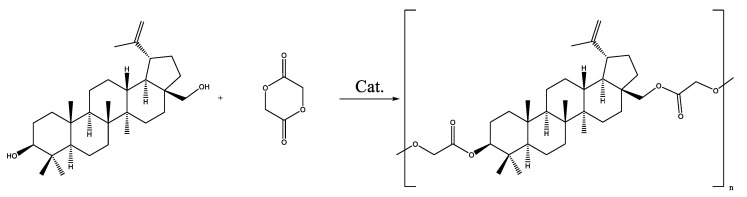
Formation of betulin copolymer with Glc.

**Figure 7:**
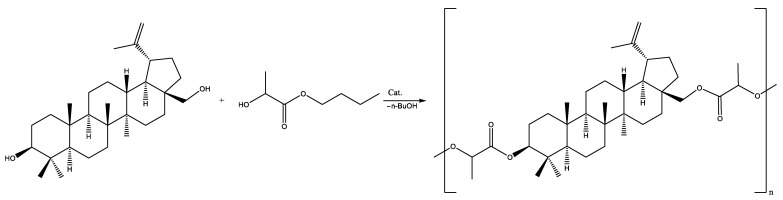
Polycondensation of betulin with BL.

The formation of a copolymer of betulin with hydroxycarboxylic acids (glycolic and lactic) was not observed. In the case of the copolymerization of betulin with LA, a LA oligomer with a molecular weight of 500 g·mol^−1^ was formed (Figure 3, Table 3). At the same time, GA did not react with betulin (Figure 4, Table 3). This may be due to the inhibitory effect of water. In order to avoid this, it was decided to use lactic and glycolic acids’ cyclic derivations—lactide (Lac) and glycolide (Glc)—in ring-opening polymerization (ROP) (Figure 5 and Figure 6). Butyl lactate (BL) was chosen for a controlled polycondensation process without water (Figure 7).

The molecular weight analysis of polymers obtained with Lac and Glc comonomers in the ROP reaction showed a better result than polycondensation (Table 3 and Table 4). At the same time, in the case of polycondensation with BL, butyl alcohol is formed as a by-product. This inhibits the negative effect of water on active centers of polymer chain growth by forming an azeotrope with it, resulting in the observed polymer formation (Table 4).

It is known that the primary hydroxyl group C-28 of betulin is more active than its secondary C-3 group. To activate the low-activity hydroxyl group of betulin (C-3 group), tert-butyl hydroperoxide (TBHP) was proposed as an initiator along with γ-Al_2_O_3_, acting as a catalyst for the copolymerization process. The beneficial effect of TBHP may be caused by the formation of an alkoxyaluminum intermediate with TBHP [23], acting as the hydrogen acceptor, thereby promoting activation of the inactive C-3 group. The effects of TBHP on the copolymerization process were assessed by varying the synthesis time and comonomers. The results are presented in Table 5.

**Figure 8:**
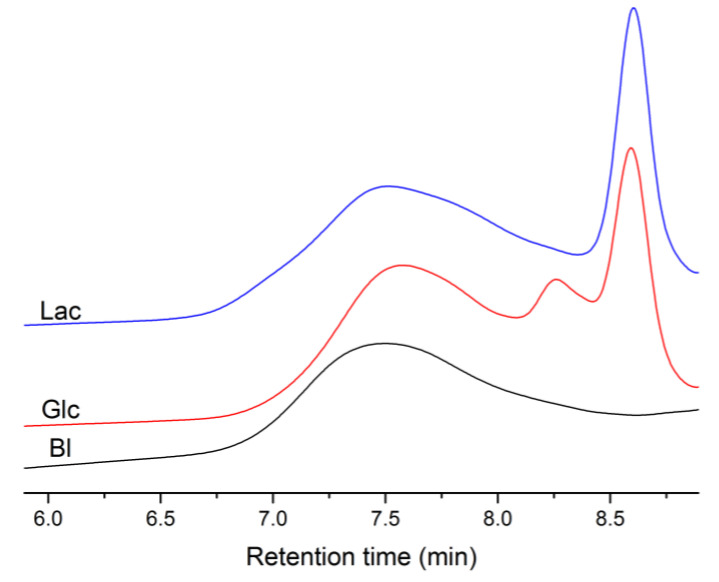
GPC chromatograms of the obtained polymers.

MW analysis showed that the catalytic system (γ-Al_2_O_3_/TBHP) allows for achieving better molecular weight increases more intensely. This effect is most effectively observed in the polycondensation process for 24 h with butyl lactate (M_n_ = 6900 g·mol^−1^). The low polymer yield when using Lac and Glc as comonomers may be attributed to the release of water as a by-product during the addition of the lactide/glycolide fragment to betulin. This water, in turn, leads to the reduction of lactide/glycolide to lactic/glycolic acid. These acids then form lower molecular weight polymers with betulin, as evidenced by GPC chromatograms (Figure 8).

**Figure 9:**
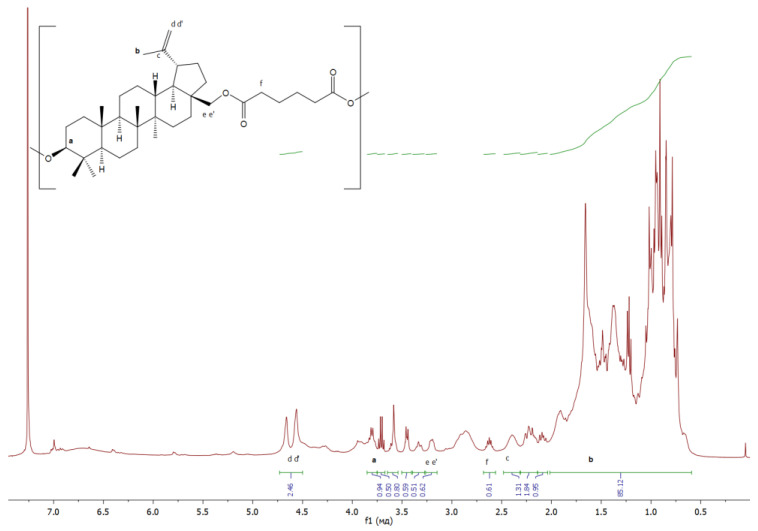
^1^H NMR spectra of copolymer based on betulin and adipic acid.

**Figure 10:**
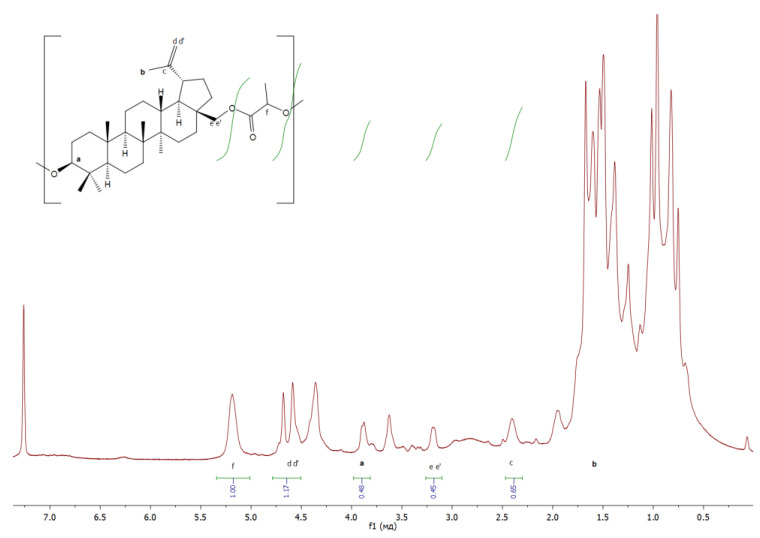
**^1^**H NMR spectra of copolymer based on betulin and lactide.

**Figure 11:**
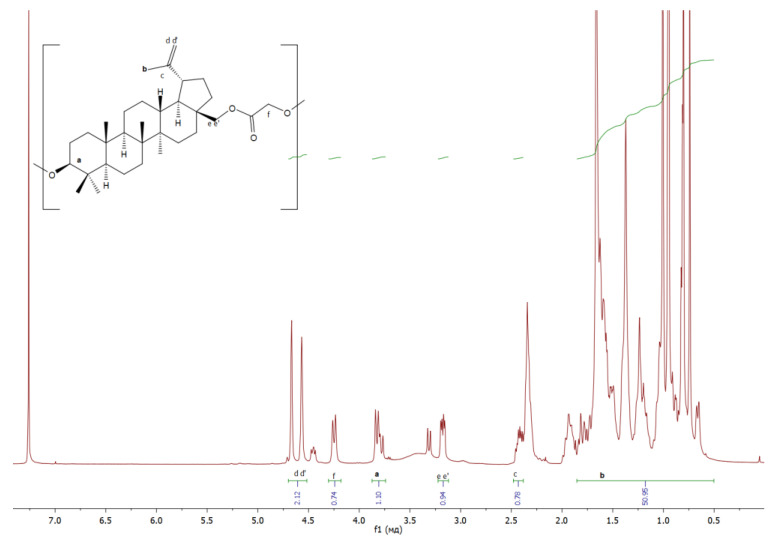
^1^H NMR spectra of copolymer based on betulin and glycolide.

**Figure 12:**
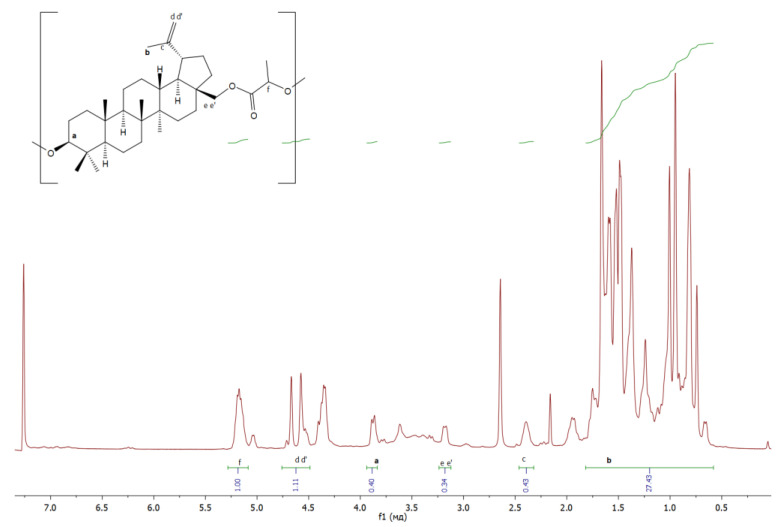
**^1^**H NMR spectra of copolymer based on betulin and butyl lactate.

The structure of the resulting polymers was confirmed by ^1^H NMR: 

**AA** (Figure 9): δ/ppm = 3.81−3,90 (betulin, a), 1.66 (betulin, b), 2.40–2.44 (betulin, c), 4.52–4.67 (betulin, dd’), 3.16–3.20 (betulin, ee’), 2.67–2.55 (adipic acid, f);

**Lac** (Figure 10): δ/ppm = 3.81−3,90 (betulin, a), 1.67 (betulin, b), 2.40–2.44 (betulin, c), 4.59–4.68 (betulin, dd’), 3.17–3.20 (betulin, ee’), 5.19–5.2 (lactic acid monomer, f);

**Glc** (Figure 11): δ/ppm = 3.81−3,90 (betulin, a), 1.66 (betulin, b), 2.40–2.44 (betulin, c), 4.52–4.67 (betulin, dd’), 3.16–3.20 (betulin, ee’), 4.24–4.26 (glycolic acid monomer, f);

**BL** (Figure 12): δ/ppm = 3.86−3,90 (betulin, a), 1.66 (betulin, b), 2.39 (betulin, c), 4.58–4.67 (betulin, dd’), 3.16–3.20 (betulin, ee’), 5.17–5.19 (lactic acid monomer, f).

Furthermore, the obtained polymers were characterized by DSC and TG methods, and their toxicity was assessed.

**Figure 13:**
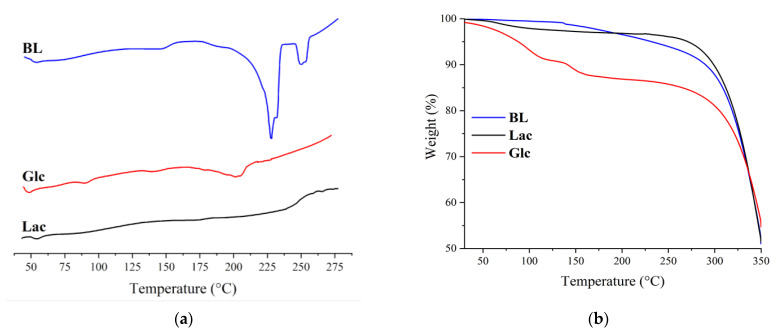
DSC (**a**) and TG (**b**) profiles of the obtained polymers.

From the DSC results (Figure 13a), it is clear that the obtained polymers are characterized by two endo-effects of glass transition and melting temperature. For the BL polymer, the values are 54 °C and 228 °C; for Glc, 48 °C and 201 °C; and for Lac, 55 °C and 227 °C, respectively. It can be observed that, based on the ratio of glass transition and melting effects, the most crystalline is the BL polymer, and all synthesized polymers are thermoplastic. It is also evident that the temperature effects of betulin-based polymers do not change, regardless of their molecular weight. This can be attributed to the rigid structure of the betulin molecule. From the TG results (Figure 13b), it is clear that the obtained polymers are not thermally resistant polyesters, showing that the melting of these polymers proceeds with oxidation in air.

**Figure 14:**
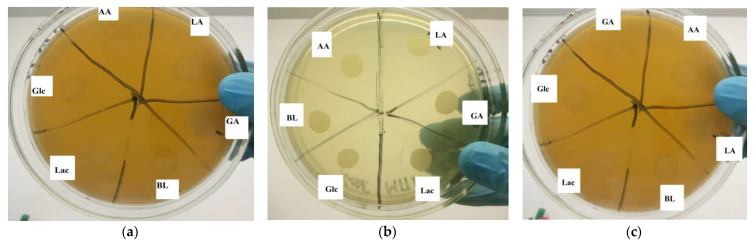
Results of a disk diffusion test for the toxicity of polymer samples to bacteria: (**a**) *S. aureus*; (**b**) *E. coli*; (**c**) *S. epidermidis* after 48 h of cultivation.

According to the toxicity study, a zone of suppression of the growth of Gram-positive bacteria (*S. aureus, S. epidermidis, L. Plantarum*) and Gram-negative bacteria (*E. coli*) was not identified. In addition, it was observed, especially for Gram-negative bacteria, that the studied polymer samples can act as a carbon source. Because the edges of the sample disks are overgrown with bacteria on top, we can conclude that the resulting polymer materials are not toxic to the studied strains (Figure 14).

**Figure 15:**
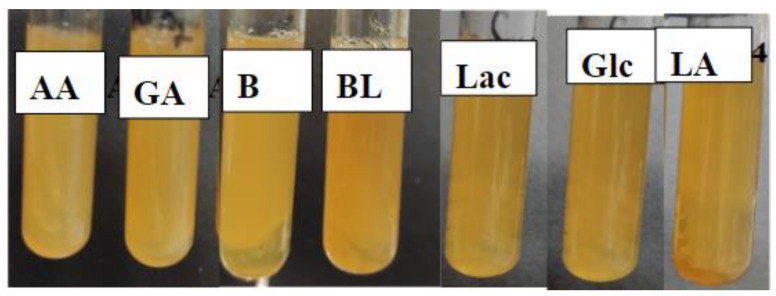
Results of the toxicity of polymer samples to *L. plantarum* after 72 h of cultivation.

When studying the influence of polymer samples on the growth of Gram-positive anaerobic non-spore-forming lactic acid bacteria, it was revealed that the growth of the culture of Gram-positive bacteria *L. plantarum* (Figure 15) is maintained.

**Figure 16:**
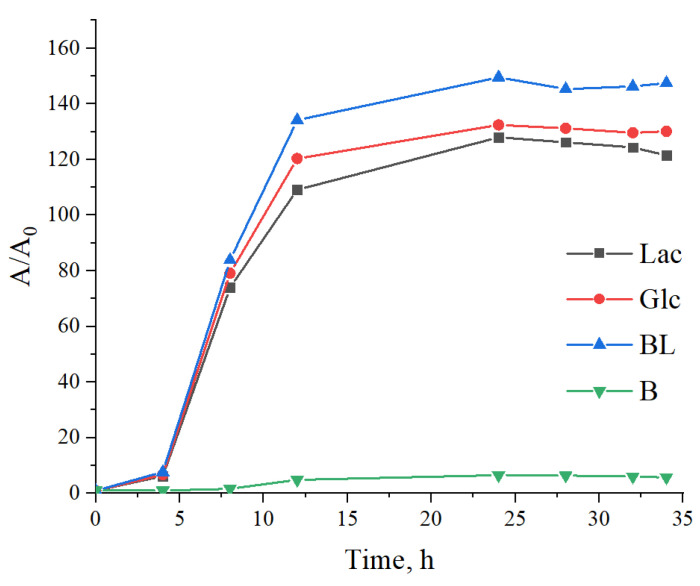
Increase in bacterial biomass of *L. plantarum* during 34 h of cultivation.

Moreover, when studying the effect of polymer samples (BL, Glc, Lac) on a culture of *Lactobacillus plantarum* in a liquid nutrient medium using spectrophotometry, a slight increase in the growth rate of the cell culture is observed. Betulin (B) was selected as a comparative sample, and its presence inhibited cell growth (Figure 16), aligning with the data obtained on cultivation in a dense nutrient medium.

Finally, the efficiency of the catalyst (γ-Al_2_O_3_) and catalytic system (γ-Al_2_O_3_/TBHP) proposed in the current study was evaluated by comparing their catalytic performance for the polycondensation of betulin with BL with the results obtained using tin octoate (Sn(Oct)_2_) and zinc acetate (Zn(OAc)_2_), which are widely used as polycondensation catalysts (Table 6).

The presented results illustrate that γ-Al_2_O_3_ and γ-Al_2_O_3_/TBHP show a catalytic performance superior to the activity of widely used copolymerization organometallic catalysts. The proposed catalytic system also demonstrates greater efficiency in terms of polymer yield. In addition, the proposed catalyst and catalytic system are non-toxic, in contrast to organometallic alternatives, making them suitable for producing polymer materials for medical purposes.

## 4. Conclusions

In light of the escalating environmental challenges arising from the persistent growth of polymer waste, coupled with an urgent need to develop new methods and approaches to the synthesis of biodegradable polymer materials, this work aimed to demonstrate the possibility of producing such materials based on betulin, organic acids and their derivatives. The primary approach prioritizes the use of renewable raw materials (biomass) while eliminating the use of toxic compounds. The catalysts employed in this study include gamma-alumina and its mixture with TBHP.

The outcomes of this study demonstrate the successful synthesis and characterization of linear thermoplastic polyesters derived from betulin and hydroxycarboxylic acid derivatives (lactide, glycolide and butyl lactate). The toxicity of these polymers towards several Gram-positive and Gram-negative bacteria was assessed. Comparative studies revealed that the molecular weight and yield of the resulting copolymer of betulin and butyl lactate exceeded the corresponding values obtained using organometallic catalysts. This demonstrates the effectiveness of the proposed catalyst/catalytic system.

Considering the renewable nature of the raw materials, the catalyst/catalytic system used and the non-toxic nature of the resulting polymers, the approach presented in this study looks promising for the future synthesis of multifunctional, biocompatible polymer materials. These materials, with their demonstrated effectiveness and potential applications, particularly in the medical field, contribute to a sustainable and environmentally friendly paradigm in polymer synthesis.

## 5. Patents

Part of the work described in this paper was the subject of a patent: Zinovyev, A.L.; Gorbunova, A.; Poletykina, E.I.; Kolobova, E.N.; Pestryakov, A.N. 2023. Method For Producing Copolymer Based on Betulin. RU Patent 2798621 C1 (Published: 23 June 2023).

## Figures and Tables

**Figure 1:**
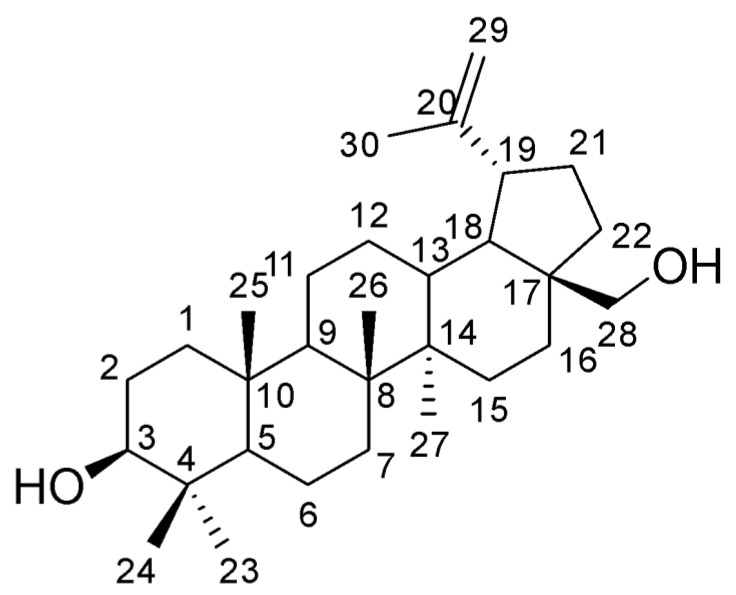
The structure of betulin.

**Table 1:** Polymer materials based on betulin.

№	Polymer Materials	Substrates, Conditions	Yield (%)	Ref.
1	Polyanhydrides	1. Synthesis of disuccinate betulin (DBB): betulin, pyridine, succinic anhydride, heat, 8 h 2. Synthesis of copolymers DBB and PEG: DBB, derivatives of PEG, acetic anhydride, 150 °C, ~ 3 h, N_2_ → vacuum	90	[4]
2	Glycopolymers	1. Synthesis of betulin-based methacrylate monomer (MBet): betulin, 4-(dimethylamino)pyridine, CH_2_Cl_2_, methacrylic anhydride, room temperature, 24 h 2. Synthesis of random and block copolymers: MBet, 6-O-Methacryloyl-1,2:3,4-di-O-isopropylidene-D-galactopyranose (MIpGa), 4-Cyano-4- [(propylsulfanylthiocarbonyl)sulfanyl]pentanoic acid (CTA), 2,2′-azoisobutyronitrile (AIBN), 75 °C, overnight/10 h	n.d.	[5]
3	Triterpene-containing polymer constructions	1. Synthesis of 28-O-maleate of betulin (MB): betulin, maleic acid, dicyclohexylcarbodiimide 2. Radical polymerization: MB and N-vinylpyrrolidone (VP) or acrylonitrile (AN) or vinyl acetate (VA) in the presence of dinitrile azobisisobasic acid (DAA), 70 °C	n.d.	[6]
4	Betulin-constituted multiblock amphiphiles	1. Synthesis of betulin-doped poly(ethylene oxide) (BEO): betulin, tetrahydrofuran, t-BuP_4_, ethylene oxide, phosphazenium salt, 45 °C 2. Synthesis of betulin-doped poly(ethylene oxide)-polyurethane (BEO-PU): BEO, toluene, isophorone diisocyanate (IPDI), diphenyl phosphate (DPP), triethylamine, 60 °C, 3 days	n.d.	[8]
5	Micro- and macroporous polyurethanes	Triphenylmethane triisocyanate in ethyl acetate, betulin in toluene, 110 °C, reflux, 1.5 h	92	[9]
6	Betulin-terephthaloyl chloride	Betulin, terephthaloyl chloride toluene, pyridine, 105 °C, 24 h	n.d.	[10]
7	Bio-based coatings	1. Synthesis of acrylated betulin (AB): betulin, THF, pyridine, acryloyl chloride, ~2 h 2. AB, acrylated epoxidized soybean oil (AESO), Darocur 1173 (2-hydroxy-2-methyl-phenyl-propanone)	n.d.	[12]
8	Aliphatic biopolyester	Betulin, aliphatic acid dichlorides (succinyl chloride, adipoyl chloride, pimeloyl chloride, azelaoyl chloride, sebacoyl chloride, dodecanedioyl chloride), pyridine, THF, N_2_, 25 °C, 15 h	85–91	[13]
9	Thermoplastic polyesters	Betulin, diacid comonomer (succinic acid (SAc), adipic acid (AAc), 1,12- dodecanedioic acid (DDDAc), 1,18-octadecanedioic acid (ODDAc)), dibutyltin oxide (DBTO), 180 °C, Ar → vacuum, 24–72 h	70–92	[14]
10	Thermosetting polyesters	Betulin, SAc, AAc or DDDAc, glycerol, dibutyltin dilaurate, vacuum at 190 °C → atmospheric pressure at 150 °C	70–92	[14]
11	Polyurethanes	Betulin, Desmodur E 22 (aromatic polyisocyanate prepolymer based on diphenylmethane diisocyanate), dibutyltin dilaurate, toluene, N_2_, 110 °C, 10–60 h	n.d.	[15]
12	Unsaturated polyesters	1. Synthesis of unsaturated oligomers: betulin, diethylene glycol, maleic anhydride, Ar, 200 °C, 1 h 2. Copolymerization: oligomer, styrene, ethyl methyl ketone peroxide, cobalt naphthenate, room temperature, atmospheric pressure	n.d.	[16]
13	Polyesters	Betulin, pimelic, azelaic or sebacic acids, N_2_ or vacuum, 256–260 °C, 22–24 h	86–97	[18]
14	Polyurethane elastomers	Betulin, castor oil, N_2_, 60-120 °C, 10h	n.d.	[19]
15	Thermosets	1. Synthesis of thermoplastic polyester: betulin, diacid, itaconic acid, dibutyltin oxide, 180 °C, Ar → vacuum, 48 h 2. Synthesis of polyester-methacrylate thermoset: thermoplastic polyester, mono-methacrylate, diphenyl(2,4,6- trimethylbenzoyl)phosphine oxide, UV	n.d.	[20]

Note: n.d.—no data.

**Table 2:** Structural and textural properties of γ-Al_2_O_3_ (data from [21]).

Sample	Observed Phase (Framework)	Specific Surface Area (S_BET_), m^2^/g	Pore Size, nm	Pore Volume, cm^3^/g
γ-Al_2_O_3_	gamma-alumina (cubic)	274	11.7	1.14

**Table 3:** Results of betulin polycondensation with AA, LA and GA.

Comonomer	M_n_^GPC^, g·mol^−1^	M_w_^GPC^, g·mol^−1^	Yield, %
AA	1600	1900	75
LA	500	500	-
GA	-	-	-

Note: M_n_^GPC^—number-average molecular weight according to the GPC; M_w_^GPC^—weight-average molecular weight according to the GPC.

**Table 4:** Results of betulin copolymerization with oxycarboxylic acid derivatives.

Comonomer	M_n_^GPC^, g·mol^−1^	M_w_^GPC^, g·mol^−1^	Yield, %
Lac	800	900	73
Glc	750	900	71
BL	500	750	96

Note: M_n_^GPC^—number-average molecular weight according to the GPC; M_w_^GPC^—weight-average molecular weight according to the GPC.

**Table 5:** Molecular weight analyses of the betulin-based polyesters obtained at different reaction times with and without TBHP.

Comonomer	Time, h	γ-Al_2_O_3_			γ-Al_2_O_3_/TBHP		
		M_n_^GPC^, g·mol^−1^	M_w_^GPC^, g·mol^−1^	Yield, %	M_n_^GPC^, g·mol^−1^	M_w_^GPC^, g·mol^−1^	Yield, %
Lac	3	800	900	73	1000	1300	76
Lac	15	1200	2200	75	1300	2300	78
Lac	24	1600	3100	75	1600	3100	78
Glc	3	750	900	71	1200	2300	72
Glc	15	1000	1400	72	1100	1600	73
Glc	24	1600	1600	71	1300	2900	75
BL	3	500	700	96	1500	1900	92
BL	15	3000	4100	95	5300	11800	98
BL	24	5600	6000	95	6900	7200	91

Note: M_n_^GPC^—number-average molecular weight according to the GPC; M_w_^GPC^—weight-average molecular weight according to the GPC.

**Table 6:** Comparison of the catalytic activity of γ-Al_2_O_3_, γ-Al_2_O_3_/TBHP and organometallic catalysts in the copolymerization of betulin with BL for 24 h.

Catalyst	M_n_^GPC^, g·mol^−1^	M_w_^GPC^, g·mol^−1^	Yield, %
without catalyst	500	500	75
γ-Al_2_O_3_	5600	6000	93
γ-Al_2_O_3_/TBHP	6900	9500	91
Sn(Oct)_2_	5500	4400	25
Zn(OAc)_2_	4300	4100	47

Note M_n_^GPC^—number-average molecular weight according to the GPC; M_w_^GPC^—weight-average molecular weight according to the GPC.

## Data Availability

Data are contained within the article.
